# Supplementary material for: The causal relationship between the human gut microbiota and pyogenic arthritis: a Mendelian randomization study
Source: Front Cell Infect Microbiol. 2024 Nov 26;14:1452480. doi: 10.3389/fcimb.2024.1452480 (PMC11629706; doi:10.3389/fcimb.2024.1452480)
Supplement: Supplementary file 1 [file DataSheet1.zip › mendelian test/ebi-a-GCST90027590.csv_forest.pdf]

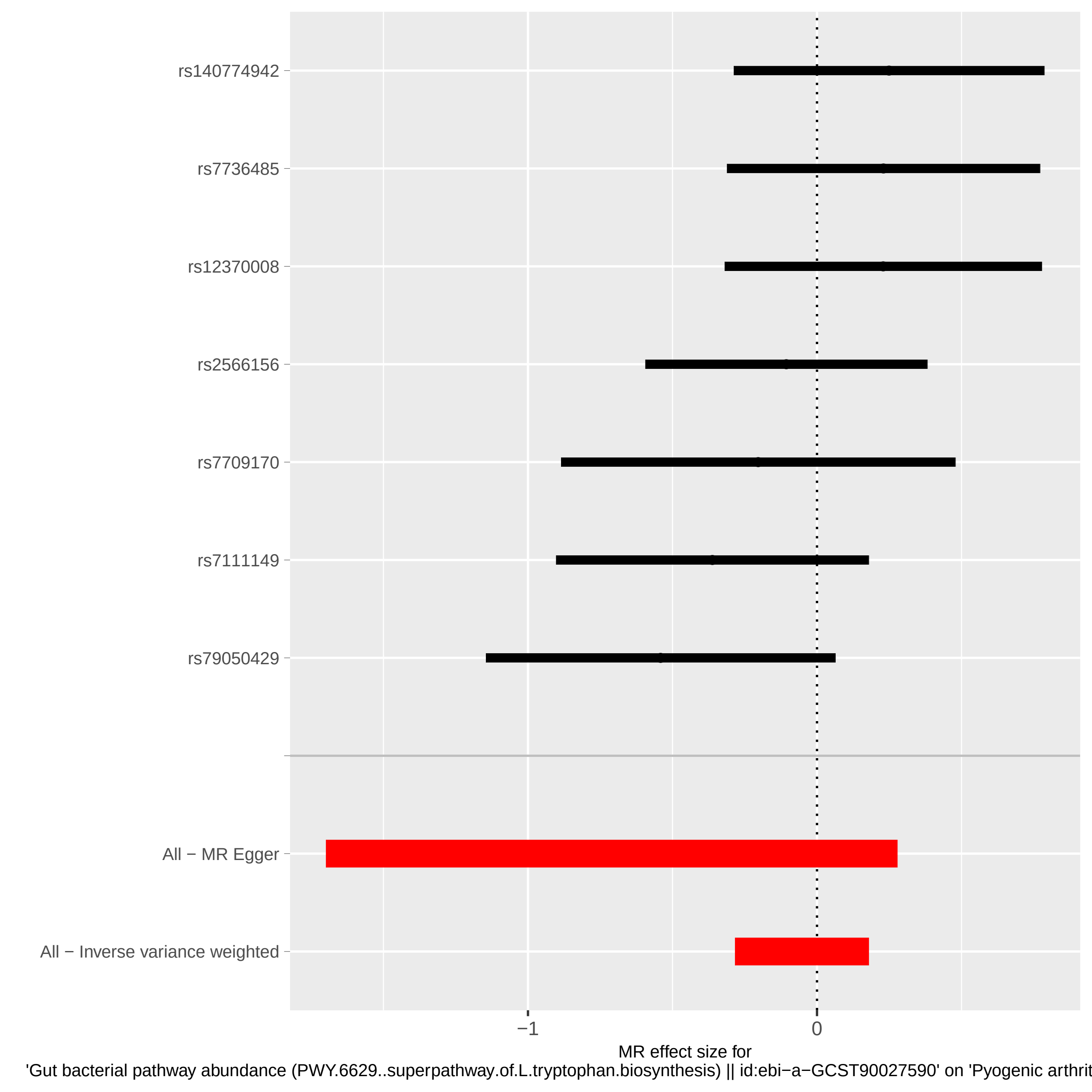
rs140774942
rs7736485
rs12370008
rs2566156
rs7709170
rs7111149
rs79050429
All − MR Egger
All − Inverse variance weighted
−1
0
MR effect size for
'Gut bacterial pathway abundance (PWY.6629..superpathway.of.L.tryptophan.biosynthesis) || id:ebi−a−GCST90027590' on 'Pyogenic arthrit
